# Development and Validation of Survival Prediction Models for Patients With Pineoblastomas Using Deep Learning: A SEER‐Based Study

**DOI:** 10.1002/cnr2.70303

**Published:** 2025-08-07

**Authors:** Xuanzi Li, Shuai Yang, Yingpeng Peng, Xueqiang You, Shunli Peng, Siyang Wang, Dasong Zha, Shuyuan Zhang, Chuntao Deng

**Affiliations:** ^1^ The Cancer Center The Fifth Affiliated Hospital of Sun Yat‐sen University Zhuhai Guang dong Province China; ^2^ Department of Radiotherapy of The Cancer Center The Fifth Affiliated Hospital of Sun Yat‐sen University Zhuhai Guang dong Province China; ^3^ School of Innovative Engineering Macao University of Science and Technology Macao Macao Special Administrative Region China

**Keywords:** artificial intelligence, deep learning, machine learning, pineoblastoma, SEER

## Abstract

**Purpose:**

Pineoblastomas (PBs) are rare central nervous system tumors primarily affecting children and adolescents, with limited data on clinical characteristics and survival outcomes. Prognosis prediction models for this disease are lacking. The purpose of this study was to develop deep learning (DL) models for predicting 3‐year survival in patients with pineoblastoma.

**Methods:**

Patients with pineoblastomas of all ages were identified from the Surveillance, Epidemiology, and End Results (SEER) database (1975–2019). Deep neural networks (DNN) were trained and tested at a ratio of 7:3 in a 5‐fold cross‐validated fashion. Multivariate CPH models were constructed for comparison. The primary outcomes were 3‐year overall survival (OS) and disease‐specific survival (DSS). All the variables were included in the analysis. Receiver operating characteristic (ROC) curve analysis and calibration plots were used to evaluate the model performance.

**Results:**

A total of 145 patients were included in this study. The area under the curve (AUC) for the DNN models was 0.92, 0.91, and 0.749 for OS and 0.76 for DSS. The DNN models exhibited good calibration: the OS model (slope = 0.94, intercept = 0.07) and DSS model (slope = 0.81, intercept = 0.20).

**Conclusion:**

Our DNN models showed a more accurate prediction of survival outcomes in patients with pineoblastoma than the widely used CPH models. These results indicate the potential of DL algorithms to improve outcome prediction in patients with rare tumors.

## 1 Introduction

Pineoblastomas (PBs) are rare and highly aggressive tumors mainly affecting children and adolescents, making up about 40% of pineal parenchymal tumors and under 0.1% of all intracranial tumors [1, 2, 3]. Classified as WHO grade 4, they often spread through cerebrospinal fluid, resulting in a poor prognosis [4]. The standard treatment for this tumor remains poorly defined. Effective management involves a combination of surgery, radiotherapy, and chemotherapy, leading to a median survival of 20–34.2 months and a 3‐year survival rate of 46.7% to 52.3% [5, 6, 7].

Due to the rarity of PBs, no clinical trials have been dedicated to this disease. Existing literature on PBs patients survival was largely case reports and small single‐center retrospective studies, which may not represent the general population [8, 9, 10, 11]. A number of scholars have tried to identify the prognostic factors for this tumor; yet its clinical significance remains debated [12, 13]. Reliable predictors and predictive models for long‐term survival of PBs are still lacking. However, accurate prognosis assessment is crucial for patient counseling and informed decision making in clinical practice. Hence, developing novel prognosis models for this rare tumor is highly urgent and warranted.

Several prognostic tools in patients with PBs have been proposed in previous studies, but the results were not ideal, with the area under the curve (AUC) of the receiver operating characteristic (ROC) curve between 0.767 and 0.802 [14, 15, 16, 17]. Additionally, the majority of models reported, such as the Cox proportional hazards model (Cox‐ph, CPH) model and nomogram, are linear in nature, relying on linearity assumptions rather than performing nonlinear analyses that reflect the real‐world clinical characteristics.

Over the past few years, artificial intelligence (AI) and big data have gradually penetrated into medicine. Machine learning (ML), particularly deep learning (DL) algorithms, is increasingly applied to solve clinical problems. A number of studies have demonstrated that DL exhibits strong performance in oncology tasks, including tumor detection and diagnosis, tumor segmentation and classification, as well as the prediction of tumor gene mutation and patient survival outcomes [18, 19]. It has been determined that these techniques can enhance the predictive accuracy of linear models by learning complex nonlinear relationships from input data [20].

The Surveillance, Epidemiology, and End Results (SEER) database is a national, publicly accessible large cancer database with longitudinal follow‐up data, providing a good source for investigating rare tumors. Here, we collected PBs patients data from the SEER database (1975–2019) and developed a DL model and CPH model to predict the 3‐year overall survival (OS) and disease‐specific survival (DSS). We hypothesized that the DL model would outperform the CPH model in terms of survival prediction.

## 2 Methods

### 2.1 Data Source and Extraction

The SEER database (http://seer.cancer.gov/seerstat), covering about 48% of the U.S. population, is a valuable source for studying rare tumors. In this study, we used SEER*Stat software (version 8.4.1) to compile case listings and select patients. The data for each case were extracted from the database (Incidence‐SEER Research Plus Data, 8 Registries, Nov 2021 Sub (1975–2019)‐Linked To County Attributes—Time‐Dependent (1990–2019) Income/Rurality, 1969–2020 Counties, National Cancer Institute, DCCPS, Surveillance Research Program, released April 2022, based on the November 2021 submission). The extracted variables included the year of diagnosis, age, sex, race, tumor size and extension, treatment modality (surgery, radiotherapy, and/or chemotherapy), and survival information (survival time and vital status at the last follow‐up). Since the SEER database data were anonymized, ethical approval or informed consent was not necessary for this study.

### 2.2 Inclusion and Exclusion Criteria

We selected patients diagnosed with pineoblastoma from 1975 to 2019 based on the International Classification of Diseases for Oncology version 3 (ICD‐O‐3) coding system. Patients were included if the following inclusion criteria were met: (a) primary tumor located in the pineal gland, (b) ICD‐O‐3 histology/behavior code = 9362/3, and (c) diagnosis confirmed histopathologically. Patients with more than one primary cancer and unavailable survival time or final status were excluded.

### 2.3 Model Development and Deployment

Based on prior research indicating the superiority of DL models over traditional linear models for survival analysis, we aimed to develop a DL model for survival prediction of PBs patients [20, 21]. All patients were randomly divided into training and testing cohorts in a 7:3 ratio. The training cohort was used to develop the model, and the testing cohort was used to evaluate the model. The model input included patient demographics, tumor information, and treatment modalities, such as age, race, sex, tumor size (cm), tumor extension, extent of surgical resection (gross total resection, subtotal resection), radiotherapy (yes/no), and chemotherapy (yes/no). The final endpoints of our study were 3‐year OS and 3‐year DSS.

As described previously [22], we first constructed a fully connected network with 8 layers and 256 nodes in each layer. Five networks were created, and each network was validated. The networks were trained using the adaptive moment estimation (Adam) optimization technique with the following parameters: learning rate = 0.0003, β1 = 0.9, β2 = 0.999, epsilon = 10–8, and weight decay = 10–4. Binary cross‐entropy is used as the loss function.

We used the same variables to construct a Multivariable Cox proportional hazards (CPH) model for comparison. Python version 3.6 (Python Software Foundation, Wilmington, Delaware, USA) and the DL framework PyTorch (version 1.4) were used to implement and train the DL models. CPH models were constructed using R version 4.3.0 (The R Foundation for Statistical Computing).

### 2.4 Model Evaluation

Models were tested using 5‐fold cross‐validation. To assess the model's performance, the ROC curve was employed, and the area under the ROC curve (AUC) was calculated. The AUC is a quantitative measure of model performance and represents the overall predictive value across all thresholds. Generally, the AUC ranges from 0 to 1. The closer the value of the AUC is to 1, the better the predictive power of the model. Furthermore, a calibration plot was drawn to compare predicted probabilities with actual observed risks, thereby evaluating the goodness‐of‐fit of the model. The calibration intercept and slope were then calculated. If the model was calibrated perfectly, then the intercept was 0 and the slope was 1.

## 3 Results

**FIGURE 1:**
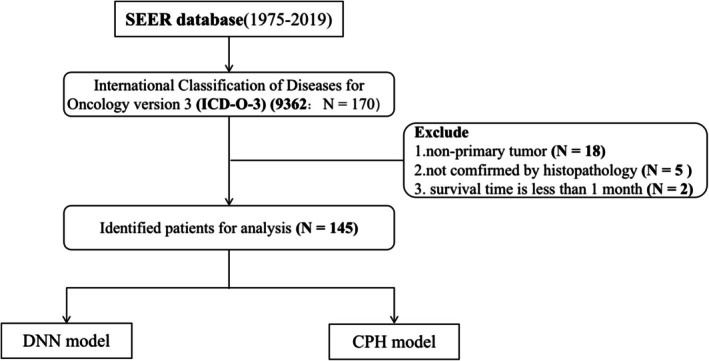
The flow chart of the data screening process from the SEER database.

The patient screening flow chart is shown in Figure 1. In this study, 145 patients were included in statistical analyses. Baseline characteristics of all patients were summarized in Table 1. The median age of the patients was 21 years. Notably, the majority of the patients were male (*n* = 75, 56.7%) and white (*n* = 103, 71%). 46.9% of the patients (*n* = 68) were children and adolescents aged between 0 and 19 years. In addition, 32.4% of the patients (*n* = 47) had tumors > 3 cm, and 11.7% (*n* = 17) had distant metastases. Among those who underwent surgery, gross total resection (GTR) was successfully achieved in 45 patients (31%). Over half of the patients (*n* = 80, 55.2%) received postoperative RT.

**TABLE 1:** The baseline characteristics of patients.

Characteristics	Total *N* = 145
Age group	
< 10 years	41 (28.3%)
10–19 years	27 (18.6%)
20–39 years	48 (33.1%)
≥ 40 years	29 (20.0%)
Gender	
Male	75 (51.7%)
Female	70 (48.3%)
Race	
White	103 (71.0%)
Black	24 (16.6%)
Asian or Pacific Islander/American Indian/Alaska Native	18 (12.4%)
Tumorsize	
< 3 cm	
≥ 3 cm	
Tumor extension	
Localized	94 (64.8%)
Regional	28 (19.3%)
Distant	17 (11.7%)
Unknown	6 (4.1%)
Months from diagnosis to treatment	
< 1 month	89 (61.4%)
≥ 1 months	51 (35.2%)
Unknown	5 (3.4%)
Surgery	
No surgery	24 (16.6%)
Local tumor destruction	14 (9.7%)
STR/PTR	50 (34.5%)
GTR	45 (31.0%)
Surgery, NOS	12 (8.3%)
Radiotherapy	
No	59 (40.7%)
Yes	80 (55.2%)
Others	6 (4.1%)
Chemotherapy	
No	73 (50.3%)
Yes	72 (49.7%)

The multivariable CPH models achieved AUC of 0.749 for OS and 0.76 for DSS. The independent prognostic factors associated with all‐cause mortality are shown in Table 2. Of note, age showed a significant influence on OS; increasing age was correlated with better clinical outcomes (10–19 years, HR = 0.20, 95% CI 0.07–0.55, *p* = 0.001; 20–39 years, HR = 0.39, 95% CI 0.18–0.81, *p* = 0.012; ≥ 40 years, HR = 0.31, 95% CI 0.11–0.84, *p* = 0.021). In addition, compared to patients with localized and regional tumors, those with distant metastasis had a worse prognosis (HR = 3.0, 95% CI 1.41–6.40, *p* = 0.004). Furthermore, chemotherapy greatly reduced the risk of mortality and prolonged the OS time (HR = 0.46, 95% CI 0.23–0.91, *p* = 0.026). No statistically significant differences were observed for any other factors.

**TABLE 2:** Multivariate CPH analysis of factors associated with all‐cause mortality.

Clinical characteristics	Overall survival (OS)			
	Hazard ratio	*P*	Confidence interval lower 95% CI upper 95% CI	
Age group				
< 10 years	References			
10–19 years	0.20	0.002	0.07	0.55
20–39 years	0.39	0.012	0.18	0.81
≥ 40 years	0.31	0.021	0.11	0.84
Gender				
Male	References			
Female	1.20	0.535	0.68	2.13
Race				
White	References			
Black	0.96	0.930	0.43	2.16
Asian or Pacific Islander/American Indian/Alaska Native	0.81	0.660	0.31	2.11
Tumor size				
< 3 cm	References			
≥ 3 cm	0.81	0.575	0.39	1.68
Unknown	1.08	0.825	0.55	2.12
Tumor Extension				
Localized	References			
Regional	0.95	0.904	0.42	2.16
Distant	3.00	0.004	1.41	6.40
Unknown	1.56	0.430	0.51	4.76
Months from diagnosis to treatment				
< 1 month	References			
≥ 1 month	0.90	0.736	0.47	1.70
Unknown	0.23	0.190	0.03	2.06
Surgery				
No surgery	References			
Local tumor destruction	0.91	0.847	0.34	2.45
PTR/STR	0.71	0.546	0.23	2.16
GTR	0.80	0.694	0.27	2.39
Surgery, NOS	2.19	0.309	0.48	9.89
Radiotherapy				
No	References			
Radiotherapy after surgery	0.55	0.149	0.25	1.23
Others	0.08	0.006	0.01	0.48
Chemotherapy				
No	References			
Yes	0.46	0.026	0.23	0.91

**FIGURE 2:**
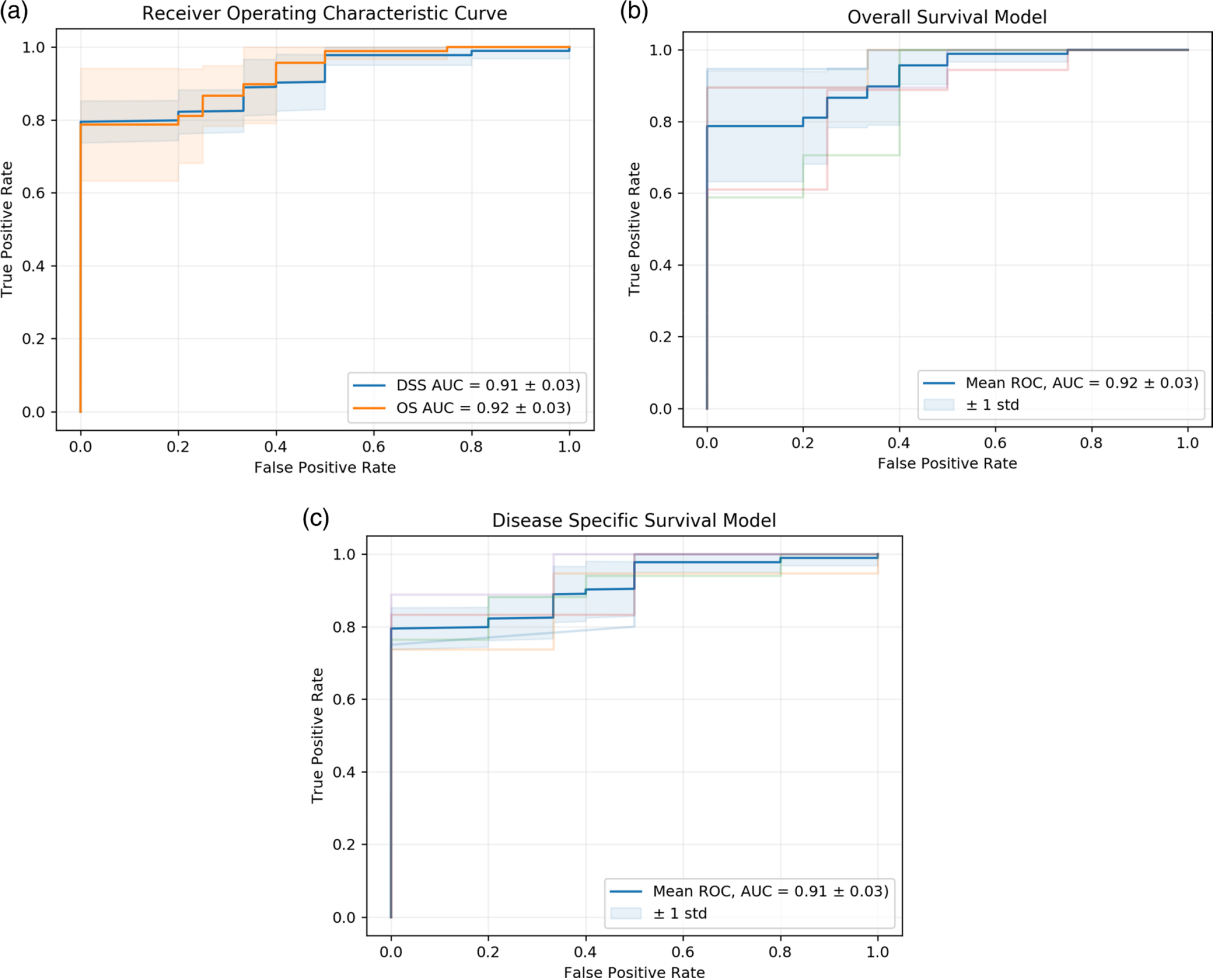
(a) Receiver operating characteristic curve of deep neural network models. (b) Receiver operating characteristic curve from each fold for the OS model. (c) Receiver operating characteristic curve from each fold for the DSS model.

**FIGURE 3:**
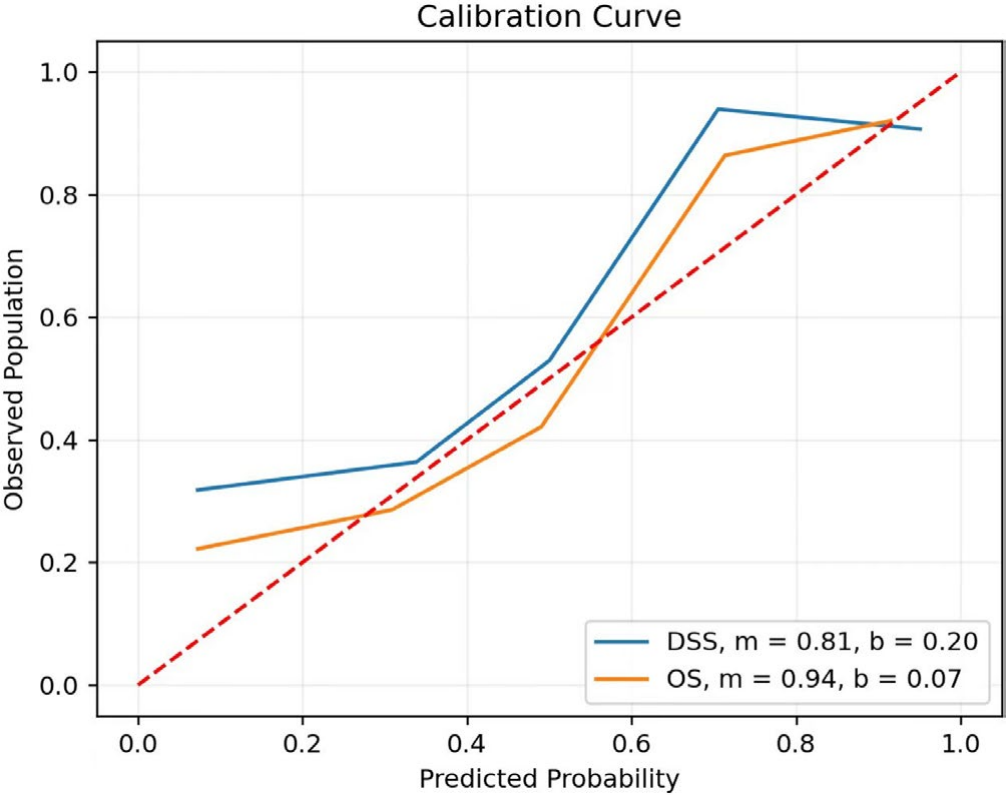
Calibration curve of deep neural network models.

In the 3‐year deep‐learning‐based OS model, the AUC value was 0.92, with a standard deviation of 0.03, across all folds (Figure 2a). The ROC plots for the OS model for each fold are shown in Figure 2b. The calibration slope of the OS model was 0.94, with an intercept of 0.07 (Figure 3).

In the 3‐year deep‐learning‐based DSS model, the AUC value was 0.91, with a standard deviation of 0.03 across all folds (Figure 2a). The ROC plots for the DSS model for each fold are shown in Figure 2c. The calibration slope of the DSS model was 0.81, with an intercept of 0.20 (Figure 3).

## 4 Discussion

In modern oncology, accurate prognostication is critically important, yet challenging for rare tumors like pineoblastoma due to the scarcity of clinical case data. Recent advances, including the increasing availability of large‐scale cancer datasets, improvements in computational performance, and the successful application of DL in oncology research, provide promising opportunities to address these challenges.

To our knowledge, this is the first study to employ a DL model for prognostication in pineoblastoma patients. Our results showed that the DL model significantly outperforms traditional Cox proportional hazards models in predicting 3‐year overall (OS) and DSS of patients with PBs. Multivariate Cox regression analysis results further identified age, chemotherapy, and distant metastasis as independent prognostic factors for all‐cause mortality.

The DL models exhibited good predictive accuracy, achieving an AUC of 0.92 for OS and 0.91 for DSS, surpassing the CPH models (AUCs 0.749 and 0.76, respectively). Additionally, the model demonstrated excellent calibration, with a near‐perfect calibration slope of 0.94 and an intercept of 0.07 for OS models, suggesting their reliability in survival estimation of PBs. In comparison to traditional linear models, a key advantage of DL models is their ability to extract multi‐layer features and discern complex non‐linear relationships between model inputs. This is achieved through a multi‐layer network structure and non‐linear activation function, thereby improving predictive accuracy [22]. This capability likely explains the superior performance of our DNN models compared to CPH models and other existing models. Prior prognostic models in PB have been limited to either pediatric or adult populations, resulting in suboptimal predictive accuray [15, 16, 17]. In contrast, our study incorporated patients across all age groups, enhancing the generalizability and reliability of our findings.

While the DL models in our study showed high predictive accuracy, their inherent “black‐box” nature presents a challenge in interpreting outputs and decision‐making processes [23]. In contrast, our CPH models provide a wealth of information on the interpretation of variables that affect outcomes. Through multivariate analysis, we identified increasing age, distant metastasis, and chemotherapy as independent prognostic factors for all‐cause mortality in pineoblastoma patients. However, it is difficult to achieve this degree of explanatory power using DNN models.

Since their emergence, DL algorithms have shown superior performance in various medical applications. While most research has focused on medical image analysis (e.g., MRI and pathological slides) [24, 25, 26], these techniques also hold great potential for tumor risk stratification and survival prediction [21, 27, 28]. Our findings corroborate this potential, demonstrating the significant superiority of DL models over traditional prognostic approaches. This suggests that DL may represent an important advancement in predicting oncological outcomes of rare tumors.

Given the infrequency of pineoblastoma, conducting large‐scale clinical trials is difficult. Current evidence primarily derives from retrospective analyses and small cohort studies, which often yield limited survival data with restricted generalizability. In this context, the SEER database provides a valuable opportunity to analyze population‐level data, facilitating the development of generalizable prognostic models and the identification of robust prognostic factors. Notably, some studies have successfully employed SEER data to develop DL models, producing promising results [29, 30, 31].

Finally, there are still some limitations in our study. First, while our DL models demonstrated strong predictive accuracy, the lack of external validation remains a key limitation. External validation is critical for assessing model generalizability and robustness, particularly in rare cancers where dataset heterogeneity may significantly impact performance. Although we utilized the SEER database—a large, population‐based registry covering approximately 48% of the U.S. population—future validation using independent, multi‐institutional cohorts is necessary to confirm clinical applicability. Second, the SEER database does not include detailed clinicopathological data such as molecular profiles, specific treatment regimens (e.g., chemotherapy protocols, radiation doses), or patient comorbidities and family history. These variables may influence survival outcomes in pineoblastoma, potentially introducing confounding effects that our models could not account for. Third, like most DL approaches, our models suffer from limited interpretability due to their inherent “black‐box” nature. While we observed strong predictive accuracy for 3‐year survival, the complex feature interactions within deep neural networks (DNNs) remain opaque, which may limit their acceptability by clinicians. Emerging explainable AI (XAI) techniques, such as SHAP (SHapley Additive exPlanations) values or attention mechanisms, could help mitigate this issue in future iterations. Despite these limitations, our findings suggest that DL‐based survival prediction using routinely collected clinical variables holds promise for pineoblastoma, a rare malignancy with limited prognostic tools. Further validation in other cohorts incorporating more clinical features will be necessary to refine these models for clinical adoption.

## 5 Conclusion

In this study, we developed a DL model that demonstrated accurate prediction of both 3‐year overall and DSS in patients with pineoblastoma. Comparative analysis revealed superior discriminative performance of our DL approach over traditional Cox proportional hazards model. These findings suggest that DL methods may facilitate accurate prognostication in patients with rare tumors.

## Author Contributions

All authors contributed to the conception and design of the study. Material preparation, data collection, and analysis were performed as previously described by Li et al. The first draft of the manuscript was written by Xuanzi Li, and all authors commented on the previous versions of the manuscript. All authors have read and approved the final manuscript. The other two authors, Xueqiang You and Shunli Peng, provided us with a lot of guidance during the revision period of the revised manuscript, so we added them to the final list of authors.

## Ethics Statement

The authors have nothing to report.

## Conflicts of Interest

The authors declare no conflicts of interest.

## Supporting information


**Data S1:** cnr270303‐sup‐0001‐Figures.docx.

**Table S1:** cnr270303‐sup‐0002‐TableS1.pdf.

## Data Availability

The data that support the findings of this study are openly available in SEER database at http://seer.cancer.gov/seerstat.
