# Supplementary material for: Development and Validation of Survival Prediction Models for Patients With Pineoblastomas Using Deep Learning: A SEER‐Based Study
Source: Cancer Rep (Hoboken). 2025 Aug 7;8(8):e70303. doi: 10.1002/cnr2.70303 (PMC12329240; doi:10.1002/cnr2.70303)

**Supplement Material**

**Figure S1a-d The receiver operating characteristic curve and calibration of CPH model for 3-year survival prediction.**

**Figure S1a The Receiver operating characteristic curve of CPH model for 3-year overall survival prediction**


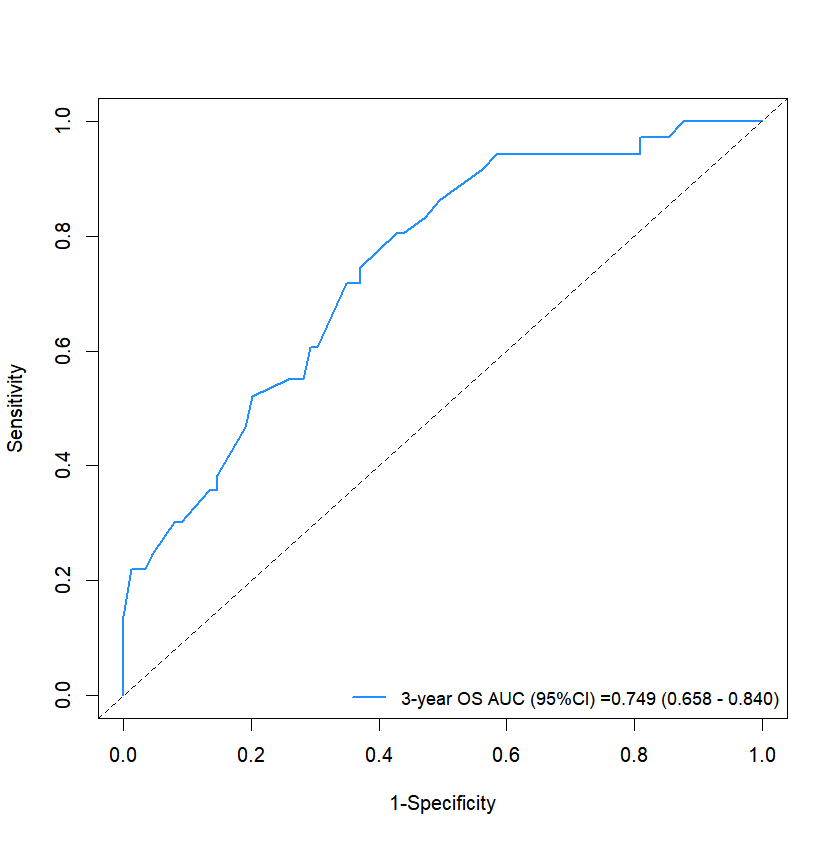


**Figure S1b The Receiver operating characteristic curve of CPH model for 3-year disease-specific survival prediction**


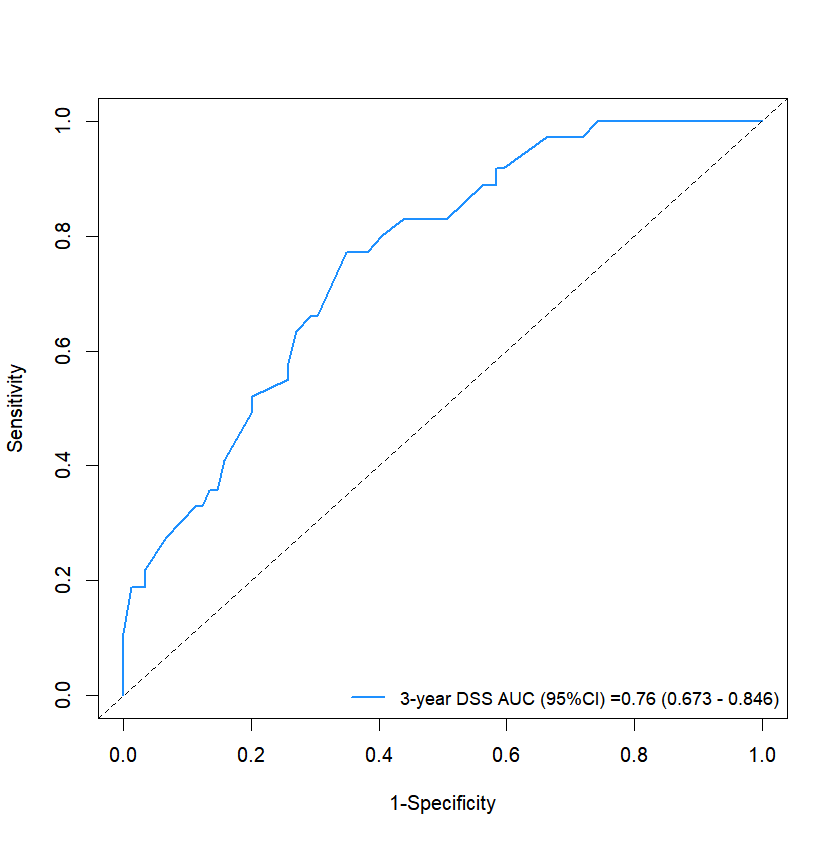


**Figure S1c The Calibration curve of CPH model for 3-year Overall survival prediction**


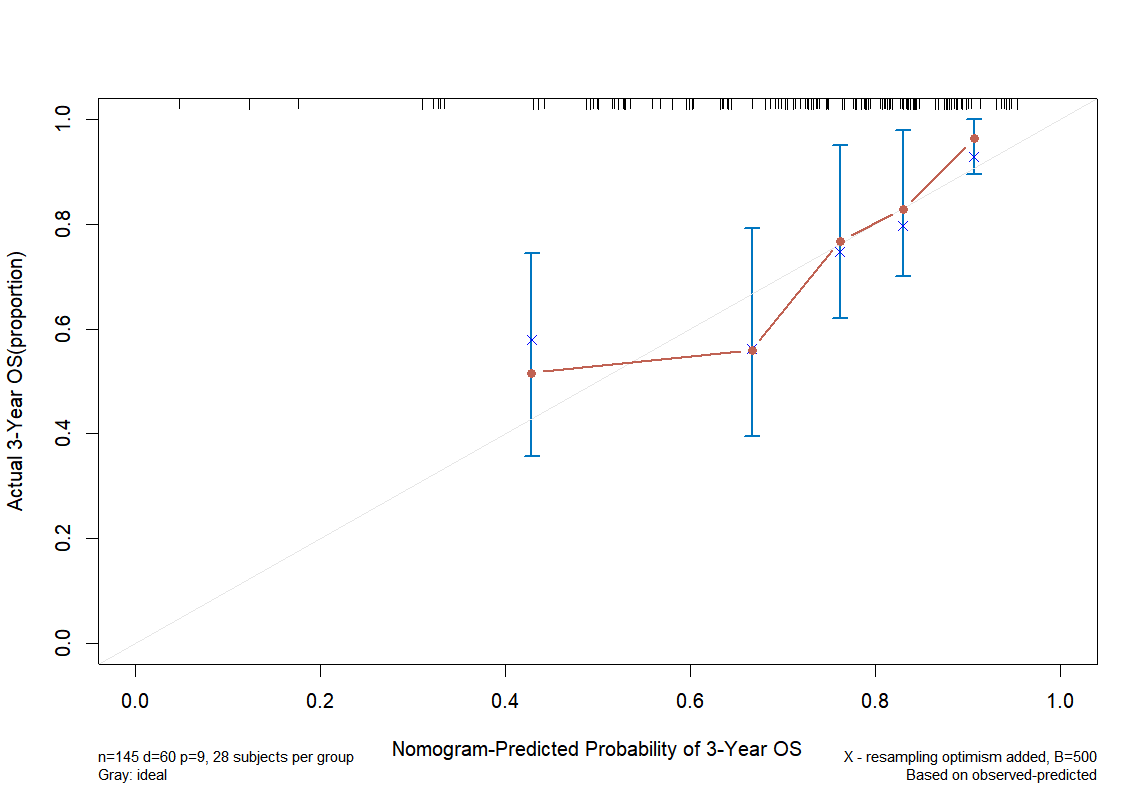


**Figure S1d The Calibration curve of CPH model for 3-year disease-specific survival prediction**


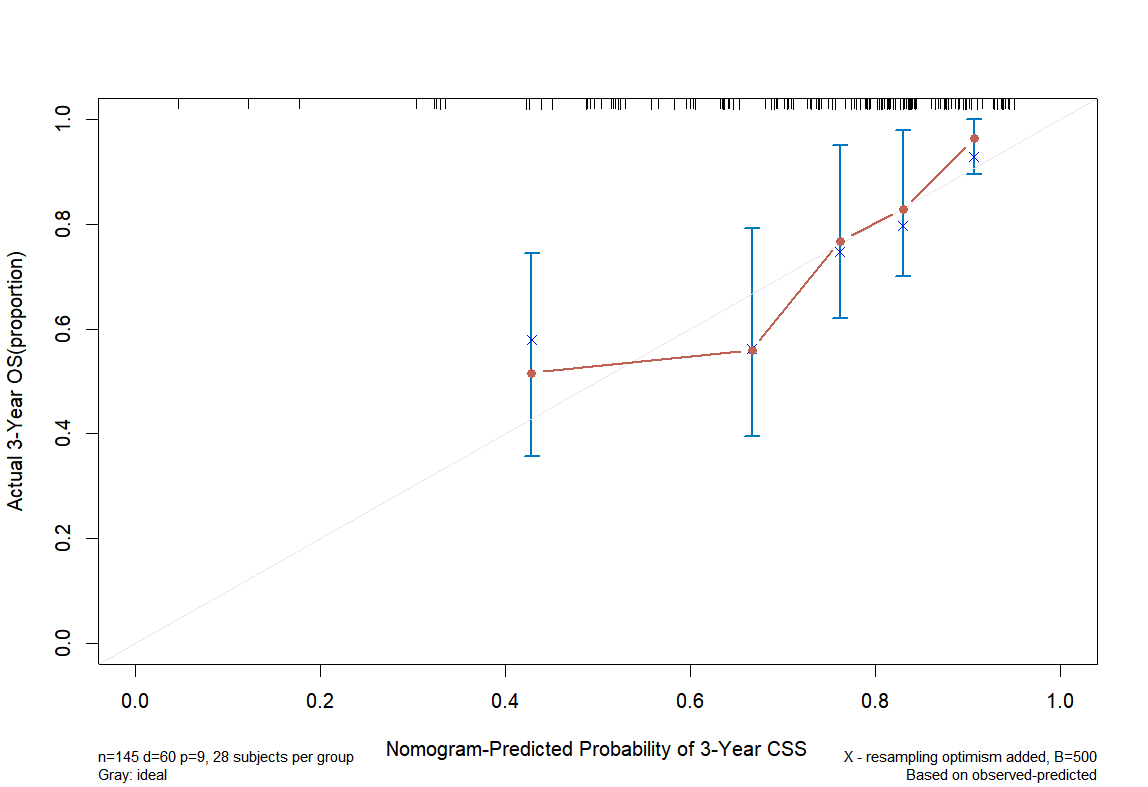


**FigureS2a-b The loss convergence graph of deep learning model**

**FigureS2a The loss convergence graph of 3-year OS deep learning model**


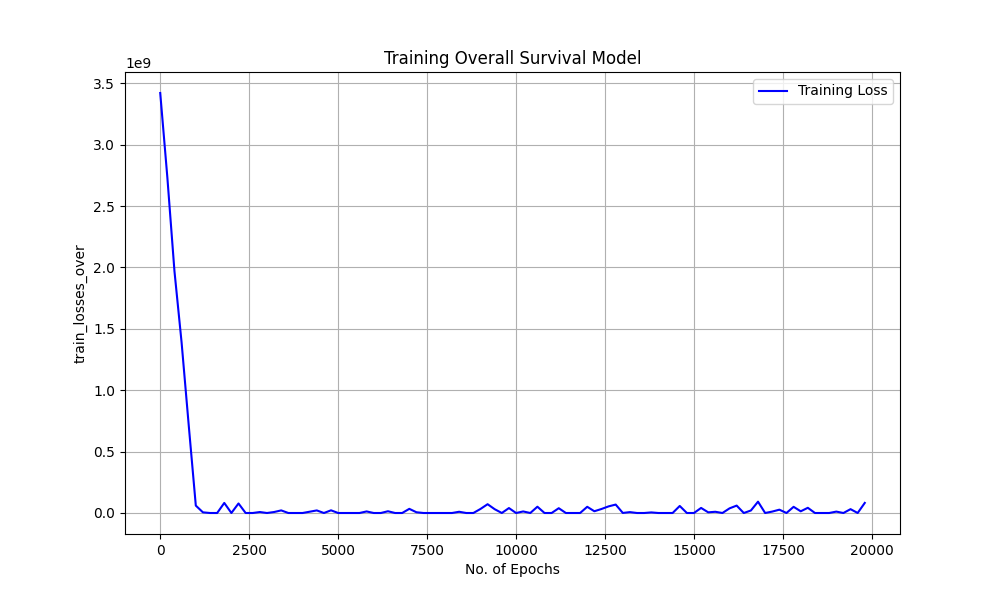


**FigureS2b The loss convergence graph of 3-year DSS deep learning model**


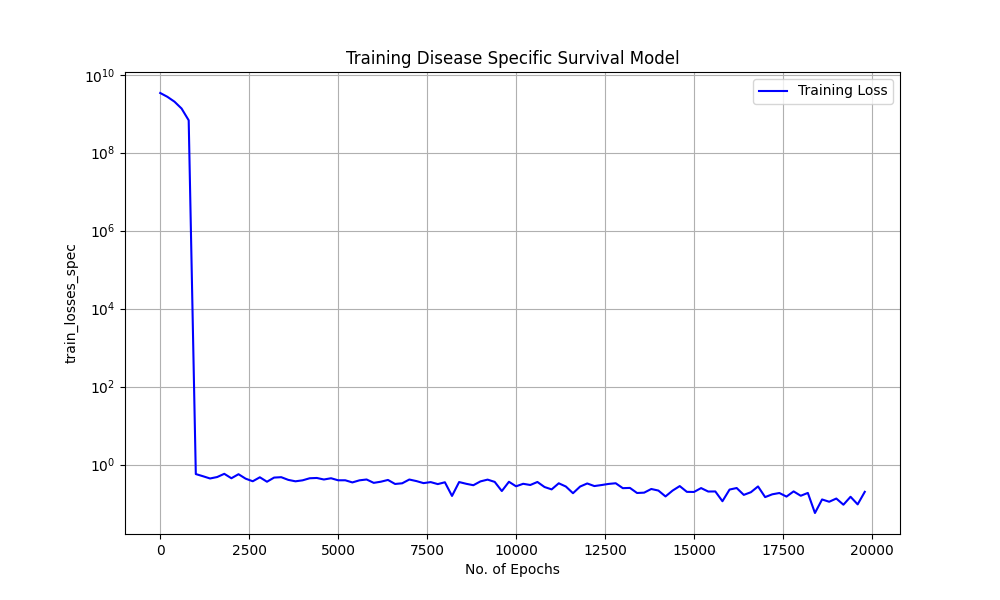

Supplement: Supplementary file 1 — Data S1: cnr270303‐sup‐0001‐Figures.docx. [file CNR2-8-e70303-s002.docx]
