# Supplementary material for: Development and Validation of Survival Prediction Models for Patients With Pineoblastomas Using Deep Learning: A SEER‐Based Study
Source: Cancer Rep (Hoboken). 2025 Aug 7;8(8):e70303. doi: 10.1002/cnr2.70303 (PMC12329240; doi:10.1002/cnr2.70303)
Supplement: Supplementary file 2 — Table S1: cnr270303‐sup‐0002‐TableS1.pdf. [file CNR2-8-e70303-s001.pdf]

TableS1 Univariate CPH analysis of factors associated with all-cause mortality

Clinical Characteristics	Overall survival (OS)			
	Hazard Ratio	P value	Confidence interval	
			lower 95%CI	upper 95%CI
**Age group**				
< 10 years	References			
10-19 years	0.26	**0.001**	0.11	0.60
20-39 years	0.71	0.237	0.40	1.25
≥ 40 years	0.57	0.130	0.28	1.18
**Gender**				
Male	References			
Female	1.12	0.637	0.69	1.82
**Race**				
White	References			
Black	1.16	0.651	0.61	2.18
Asian or Pacific Islander/American Indian/Alaska Native	0.77	0.557	0.33	1.81
**Tumor Size**				
< 3cm	References			
≥ 3cm	0.73	0.359	0.37	1.43
Unknown	1.47	0.180	0.84	2.57
**Tumor Extension**				
Localized	References			
Regional	0.84	0.614	0.42	1.68
Distant	2.42	**0.006**	1.28	4.58
Unknown	2.03	0.139	0.80	5.18
**Months from diagnosis to treatment**				
< 1 month	References			
≥ 1 month	1.09	0.737	0.65	1.85
Unknown	0.32	0.264	0.04	2.35
**Surgery**				
No surgery	References			
Local tumor destruction	1.49	0.372	0.62	3.55
PTR/STR	0.83	0.631	0.40	1.75
GTR	0.75	0.463	0.35	1.62
Surgery, NOS	1.53	0.337	0.64	3.65
**Radiotherapy**				
No	References			
Radiotherapy after surgery	0.62	0.064	0.38	1.03
Others	0.97	0.950	0.34	2.78
**Chemotherapy**				
No	References			
Yes	0.91	0.692	0.56	1.47
